# New advances in immune mechanism and treatment during ocular toxoplasmosis

**DOI:** 10.3389/fimmu.2024.1403025

**Published:** 2024-05-10

**Authors:** Zijian Chen, Shizhou Cheng, Xiaoming Chen, Zuhai Zhang, Yanhua Du

**Affiliations:** ^1^ Department of Ophthalmology, The First Affiliated Hospital of Yangtze University, Jingzhou, Hubei, China; ^2^ Physical Examination Department, The First Affiliated Hospital of Yangtze University, Jingzhou, Hubei, China

**Keywords:** toxoplasmosis, ocular toxoplasmosis, immune response, cytokines, therapy

## Abstract

Ocular toxoplasmosis (OT) is an intraocular infection caused by the parasite *Toxoplasma gondii.* OT is manifested as retinal choroiditis and is the most common infectious cause of posterior uveitis. Invasion of the retina by *T. gondii* leads to disruption of the blood-ocular barrier and promotes the migration of immune cells to the ocular tissues. Cytokines such as IFN-γ and IL-1β are effective for controlling parasite growth, but excessive inflammatory responses can cause damage to the host. In this review, we will discuss in detail the latest advances in the immunopathology and treatment of OT.

## 1 Introduction

*Toxoplasma gondii* is an intracellular apicomplexan protozoan parasite that attaches to the cell membrane via its apical complex and invades all nucleated cells of vertebrates by gliding motility (1). Toxoplasmosis is an infectious disease caused by *T. gondii* and is highly prevalent worldwide. Most primary *T. gondii* infections are asymptomatic (2), which makes early detection and treatment challenging in clinical practice. *T. gondii* comprise 3 major clonal lineages, namely type I, II and III (2, 3). Type I *T. gondii* is associated with severe OT, whereas type II strains are less virulent but are the most common cause of human infections (4). Type III strains are least virulent and are often found in domesticated and wild animals and less commonly in humans. Regardless of type, *T. gondii* can cause life-threatening disease in immunocompromised or immunosuppressed individuals, including those with HIV/AIDS (5). Additionally, these populations have a higher risk of recurrence due to reactivation of latent infection (6). Furthermore, infection during pregnancy can lead to severe neurological damage and even death in the fetus (7).

*T. gondii* invasion triggers a series of immune responses such as the release of various cytokines that are essential for defending the host against the parasite. The process by which *T. gondii* invades the eye is complex and involves the migration of the parasite across the blood-retina barrier into the retina, often resulting in infectious uveitis and other ocular complications. Therefore, early diagnosis of systemic toxoplasmosis is important for the treatment and prevention of OT and its complications. OT is typically self-limiting and frequently overlooked. However, its high recurrence rate underscores the importance of a safe and effective intervention. Current treatments (ethylaminopyrimidine and sulfadiazine) are ineffective for eradicating *T. gondii* and can cause multiple side effects, which may worsen the health of patients with compromised systemic conditions. In fact, adverse reactions to toxoplasmosis treatments have been reported to be the cause of treatment discontinuation in up to 40% of HIV patients (8).

In this review, we will discuss the systemic and ocular immune responses elicited by *T. gondii* infection, identify the limitations of current toxoplasmosis treatments, and provide insights into the development of novel therapeutic agents from an immunological standpoint.

## 2 *T. gondii* infection and dissemination

**Figure 1:**
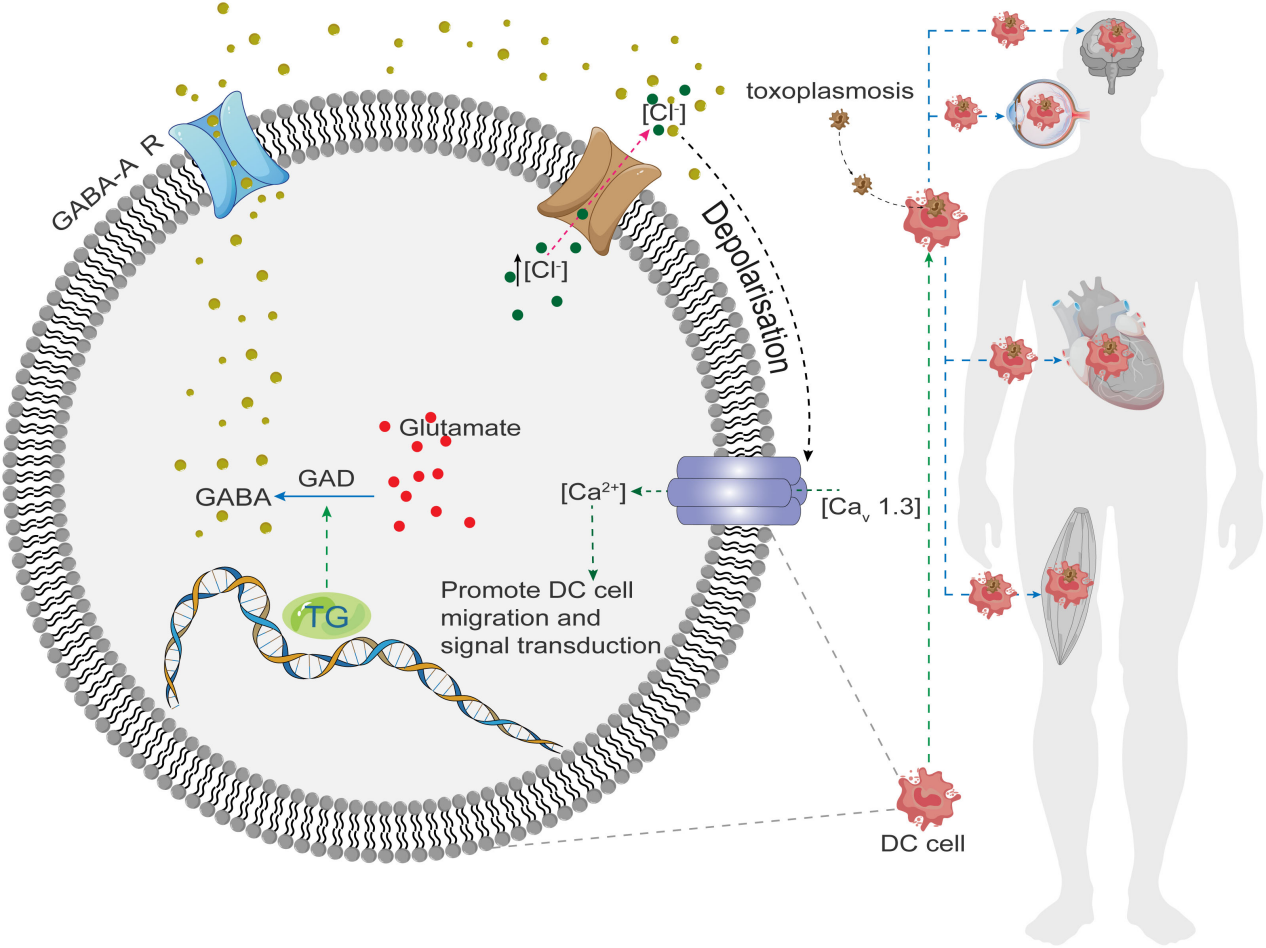
Toxoplasma gondii invades host cells and stimulates GABA synthase (GAD) to synthesize GABA, which binds to GABA-R on the membrane leading to the excretion of Cl- from the cell, generating a depolarization, which opens the downstream voltage-gated Ca2+ channel (Cav1.3), and Ca2+ inflows into the cell, where it plays the role of a second messenger to promote cell motility, migration, and signaling, and In turn, it spreads throughout the body, invading the brain, heart, muscles, and eyes.

When an intermediate host consumes raw or undercooked meat containing *T. gondii* tissue cysts, the cysts rupture as they pass through the gastrointestinal tract, releasing bradyzoites. These bradyzoites infect the host intestinal epithelium and differentiate into tachyzoites that rapidly replicate and disseminate throughout the body (9). The most accepted hypothesis for the mode of *T. gondii* transmission within the host is the “Trojan horse” mechanism. In this mechanism, the parasite invades immune cells, especially dendritic cells (DC), and exploit their mobility to disseminate. It has been shown that *T. gondii*-infected DC cells synthesize and secrete the neurotransmitter γ-aminobutyric acid (GABA), which activates GABA-A receptors (10) and induces a hypermigratory phenotype in the cells, ultimately promoting parasite transmission (**Figure 1**). In addition, the parasite can spread through the bloodstream within monocytes (11, 12) and alter their phenotype to enhance migration (13).

## 3 Initial host immune responses

### 3.1 Cellular immunity

IFNγ-mediated macrophage activation and CD8^+^ T cell-mediated cytotoxicity play important roles in controlling *T. gondii* infections. CD8^+^ T cells are essential for mediating resistance against *T. gondii* in mice by stimulating the production of endogenous interferon-γ (IFN-γ) within the first few days of infection (14, 15). *T. gondii* rhoptry protein (ROP) activates the signal transducers and activators of transcription (STAT)3/6, which modulates intracellular signaling pathways and triggers the production of host IFN-γ and IL-12. In rodents, cellular immune responses mediated by DC cells, T cells, natural killer (NK) cells, macrophages, and cytokines (IL-12 and IFN-γ) are essential for overcoming primary infection and establishing control over latent chronic infection (16). Furthermore, *T. gondii* infection can induce cell pyroptosis and IL-1β and IL-18 release by activating NLRP1 inflammasomes (17–19).

### 3.2 IL-12-mediated immune responses

Mouse experiments have demonstrated that *T. gondii* induces IL-12 production via several pathways: (1) Recognition of *T. gondii*-derived profilin-like molecules by TLR11 induces IL-12 release in a myeloid differentiation primary response protein 88 (MyD88)-dependent manner (20); (2) Recognition of profilin-like proteins by TLR12 homodimers or TLR11/TLR12 heterodimer leads to IL-12 production; (3) Detection of *T. gondii* procyclin-18 (TgCyp18) by the chemokine receptor CCR5 stimulates IL-12 expression by DCs (21); (4) *T. gondii*-mediated TLR9 activation plays a central role in coordinating DC-mediated IL-12 production and subsequent IFN-γ secretion by CD4^+^ T cells (22).

It has been shown that injection of CD8(^+^) DCs with soluble *T. gondii* antigen leads to IL-12 production in mice (23, 24). IL-12 activates CD4^+^ T cells to secrete IFN-γ, which induces the expression of inducible nitric oxide synthase (iNOS) and the generation of nitric oxide (NO), leading to cytotoxic disruption of *T. gondii* vesicles. Khan et al. found that treatment of chronically infected hosts with antigen-specific CD4^+^ T cells restored CD8^+^ T cell function and prevented reactivation of latent infection (25). Furthermore, antigen-specific CD8^+^ T cells effectively eliminated *T. gondii* cysts from immunodeficient animals (26).

### 3.3 Indoleamine 2,3-dioxygenase

When an organism is infected with *T. gondii*, binding of IFN-γ, tumor necrosis factor-α (TNF-α), lipopolysaccharide (LPS), cytotoxic T-lymphocyte-associated protein-4 (CTLA-4), CD80/CD86, and other pro-inflammatory cytokines to epithelial cells, DCs, and macrophages can induce the expression of indoleamine-2,3-dioxygenase (IDO) (27, 28). IDO is an intracellular enzyme that inhibits *T. gondii* growth (27). However, metabolites from the IDO-mediated kynurenine pathway (KP) have been shown to inhibit immune responses by suppressing CD8^+^ T cell, NK cell, DC, and macrophage functions, promoting Th2 cell and regulatory T cell (Treg) differentiation, as well as inducing the production of transforming growth factor-β (TGF-β) (27). Therefore, IDO expression must be finely regulated to enhance immune clearance of *T. gondii* while minimizing immunosuppression.

## 4 Migration to immune privileged regions of the eye

### 4.1 Migration of *T. gondii* to the retina

**Figure 2:**
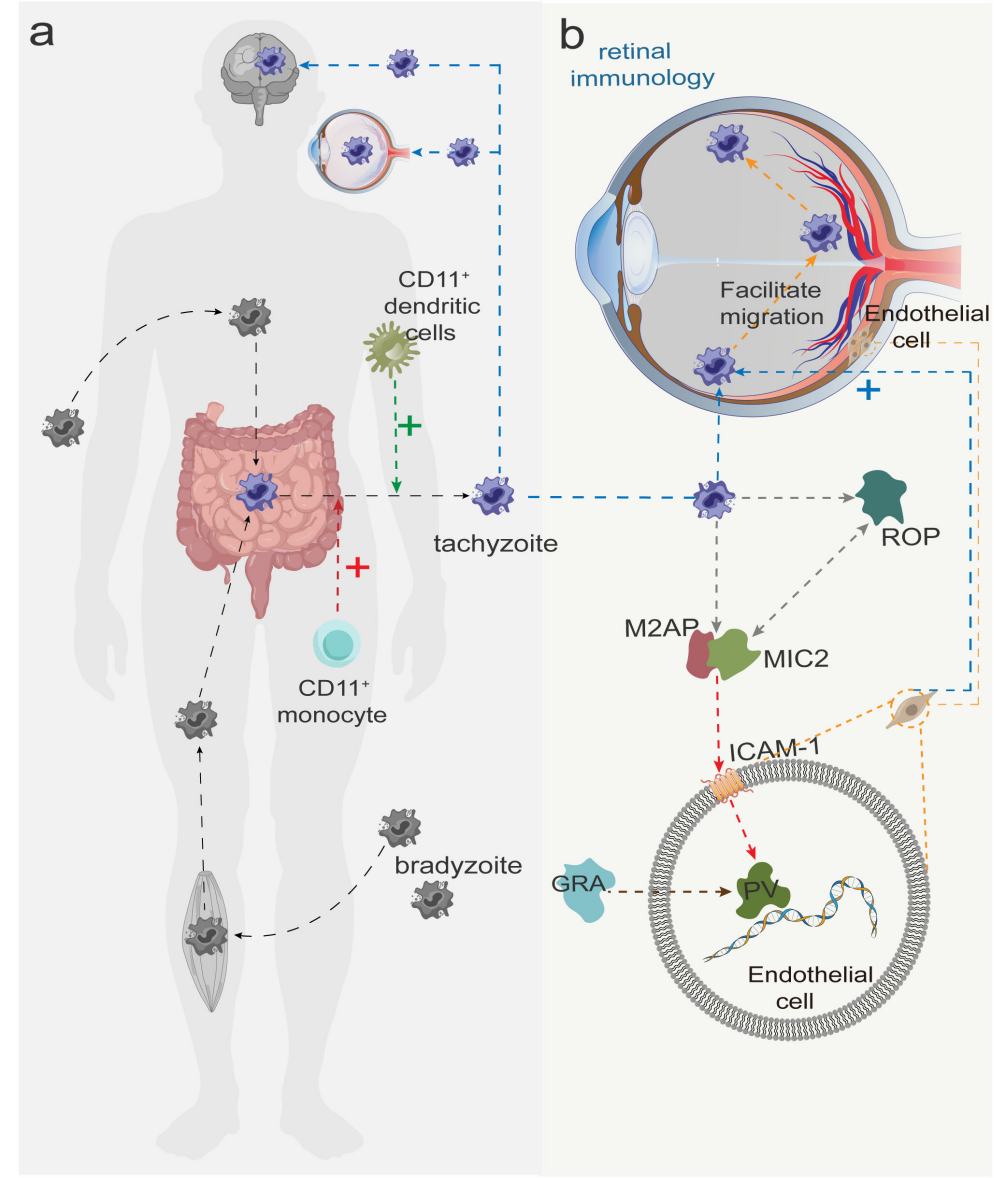
**(A)** Slow colonizers (dormant form of Toxoplasma gondii) are transmitted after invasion of the human body and are converted to tachyzoites in the small intestine. CD11+ dendritic cells and CD11+ monocytes facilitate the transport of tachyzoites to the brain and the retina (crossing the blood-brain and blood-eye barriers), with the red and green plus signs indicating facilitation. **(B)** After tachyzoites arrive at the retina with blood, MIC2 is first secreted from the tachyzoite tip and forms a MIC2-M2AP hexameric complex with MIC2-associated protein (M2AP) in a ratio of 1:1, recognizing the intercellular adhesion factor ICAM-2 on the host cell membrane. Subsequently, rod protein (rhoptry protein, ROP) is released and interacts with MIC2 to invade retinal endothelial cells and form parasitophorous vacuole (PV). Finally, dense granule protein (GRA) begin to be secreted, modifying the PV and facilitating the worm’s access to nutrients necessary for survival and replication.

In most cases, *T. gondii* remains dormant and inactive in the host without eliciting an immune response (29). Upon entry into the human body, *T. gondii* are transformed into rapidly dividing tachyzoites in the small intestine. These tachyzoites infect most nucleated cells, triggering inflammatory responses that result in immune-mediated tissue damage (30). It has been reported that CD11^+^ DCs and CD11^+^ monocytes transport the parasite to brain tissues and the retina through the blood-brain barrier and blood-retina barrier, respectively (12, 31, 32) (**Figure 2A**). Alternatively, *T. gondii* may also reach the retina through direct migration of tachyzoites across the retinal vascular endothelium (33). This migration is partly dependent on the interaction between intercellular adhesion molecule-1 (ICAM-1) on retinal endothelial cells (34) and micronemal protein 2 (MIC2) on tachyzoites (35) (**Figure 2B**). *T. gondii* infection of brain endothelial cells has been shown to upregulate ICAM-1 expression and thereby facilitate leukocyte migration across the endothelial barrier (36). In addition to ICAM-1, vascular cell adhesion molecule-1 (VCAM-1), activated leukocyte adhesion molecule (ALCAM), and chemokines CXCL21 and CXCL10 are also involved in *T. gondii* migration across the retinal vascular endothelium (32).

### 4.2 Disruption of the blood-retina barrier

The inner blood-retina barrier (iBRB) is comprised of retinal endothelial cells, pericytes, Müller cells, and astrocytes. Choroidal vessels are separated from photoreceptor cells by the retinal pigment epithelium (RPE), which together with the Bruch’s membrane, form the outer BRB (oBRB). Entry into the retina through the choroidal layer requires the parasite to cross the oBRB. Song et al. found that human monocyte THP-1 cells infected with *T. gondii* can migrate across human RPE ARPE-19 cells. Infected monocytes disrupted the oBRB through focal adhesion kinase (FAK) signaling and partly through CXCL8 (37).

### 4.3 Retinal immune responses

Müller cells and astrocytes are two types of macroglial cells found in the retina of many vertebrates, including mammals, and are essential for maintaining the homeostasis and neural organization of the retina. Müller cells extend from their apical end at the outer limb membrane (OLM) to their basal end at the inner limiting membrane (ILM). Astrocytes are primarily located in the nerve fiber layer (NFL) and ganglion cell layer (GCL). These cells secrete prostaglandins (PGE), NO, and arachidonic acid (AA), as well as molecules that stabilize the tight junctions between vascular endothelial to protect neurons from potential inflammatory damage. Furtado et al. showed that tachyzoites travel to the eyes via the bloodstream and preferentially infect Müller cells and astrocytes in the retina (38). It is worth noting that bradyzoites are present as cysts in the inner layers of the retina and can infect both glial and neuronal cells (39). The form that *T. gondii* adopts can be influenced by variations in the host eye or the virulence of the strain. Therefore, further studies are warranted to examine how these factors impact the infectious stage of *T. gondii*.

Studies have shown that high extracellular levels of TNF-α exacerbate astrocyte-mediated inflammation and neurodegeneration. Molecules from inflammatory cells, platelets, and plasma can activate Müller cells to secrete a wide range of inflammatory mediators such as TNF-α, interleukins (IL), interferons, and ICAM-1. Müller cells can directly mediate cytotoxic effects by upregulating TNF-α or monocyte chemotactic protein 1 (MCP-1) expression (40, 41), causing further damage to the retina.

Lie et al. documented increased expression of various immune molecules in the infected RPE, including BIRC3, CCL2, CXL3, CXL8, ICAM-1, IL1RN, IL-6, IL-17RB, LRP8, and NF-κB1 (42). Retinal infection by *T. gondii* induces VEGF expression in the RPE via activin-like kinase 4 (ALK4) and hypoxia-inducible factor-1 (HIF-1). Upon injury, Müller cells release high levels of VEGF, which may disrupt the BRB and potentially facilitate increased *T. gondii* migration (40). Furthermore, VEGF production by reactive astrocytes can exacerbate disease progression by increasing vascular permeability, promoting neovascularization, and contributing to cytotoxicity and secondary damage to nearby neurons and glial cells (41).

### 4.4 Intricate balance between inflammatory and regulatory cytokines

IL-10 is a potent immunomodulatory cytokine and its deficiency increases the susceptibility to *T. gondii* infection in mice. IL-10 has been shown to inhibit *T. gondii*-induced inflammation (43) by suppressing Th1 cell differentiation (44), whereas IL-10 deficiency leads to 4- to 6-fold increase in IL-12 and IFN-γ (43, 45). Thus, IL-10 plays a key role in dampening inflammation and restricting excessive proinflammatory response. Previous studies have shown that both IL-27 and IL-33 expression is critical for IL-10 production by effector T cells in *T. gondii* infection models (46, 47).

Previous study has shown that IL-6-deficient mice infected with T. gondii have severe retinal inflammation and high parasite load, indicating that IL-6 plays a key role in protecting the retina from *T. gondii* infection (48). In contrast, a study by Rochet et al. demonstrated that topical application of IL-6 antibodies significantly improved retinal structure and reduced parasite load, suggesting that IL-6 can induce retinopathy in mice (49). Similarly, *T. gondii* infection upregulates IL-17 expression, and IL-17 neutralization is partially protective against fatal *T. gondii*-associated inflammation (43, 50). IL-17 is secreted by astrocytes during acute inflammation and is protective against neural cell apoptosis and tissue damage in active uveitis by maintaining homeostasis and inhibiting intracellular calcium increase (51). However, excess IL-17 can also lead to unwanted tissue damage (43). Therefore, inflammatory cytokines must be used with caution in the treatment of toxoplasmosis as they may hinder parasite eradication and exacerbate patient discomfort.

Neutrophils play an opposite role to CD8^+^ T cells during *T. gondii* infection. Studies have shown that RPE cells infected with T. gondii can activate neutrophils via GM-CSF, IL-6 and IL-18 to produce reactive oxygen species, TNF-α and IL-1β, which are highly damaging to the retinal tissues (52, 53).

In summary, the balance of various cytokines plays a crucial role in toxoplasmosis. Certain cytokines (e.g. IL-1β, IL-6, and IL-17) can act as double-edged swords that protect the host against *T. gondii* infection but also cause deleterious effects on host cells. Consequently, this delicate balance poses challenges in the development of anti-*T. gondii* drugs, as any disruption may negatively impact patient outcomes (**Table 1**).

**Table 1:** The role of inflammatory factors in toxoplasmosis.

Inflammatory factor	Source	Effect	Refs.
IL-1β	macrophage	Induced inflammation	(17, 52)
IL-6	Damaged cells, tissue damage	Promotes body defense, but sustained synthesis has a pathological effect on chronic inflammation.	(48, 49)
IL-10	Ly6ChighCCR2+ Monocytes, B Cells, Foxp3+ CD4+ Treg cells	Inhibit some of the inflammation inducedby Toxoplasma gondii. such as Th1.	(43–45)
IL-12	CD8(+) DC, Macrophage, B cells	Activation and stimulation of NK cells, CD4 T cells and proliferation of CD8 T cells.	(20–24)
IL-17	CD4, CD8 T-cells, γδ T cells	Enhances mucosal barrier function, Recruitment of neutrophils, Promotes the production of IFNγ and IL-10.	(43, 50, 51)
IL-18	Damaged cells, tissue damage	Induction of CCL3 production Promotes IFN-γ production by NK cells	(18, 19)
IL-27	myeloid cell lineage	Induction of blimp1 and IL-10 production	(46)
IL-33	Damaged cells, tissue damage	Induction of CCL3 production Induction of IL-10 production by macrophages	(47)

## 5 Ocular immune responses and clinical manifestations

### 5.1 *T. gondii*-induced eye damage

OT is a leading cause of infectious uveitis, which can lead to visual impairment and blindness (54). OT accounts for 30–50% of all cases of posterior uveitis and may recur in 40–79% of patients (55). *T. gondii* infection during pregnancy may lead to miscarriage or congenital toxoplasmosis. In contrast to congenital toxoplasmosis, toxoplasmosis infection acquired after birth is responsible for most OT cases (56). OT is typically asymptomatic in young children (57). However, patients over 45 years of age with active lesion have a 10-fold higher risk of visual impairment. The location of the lesion is critical as macular involvement can result in 8.95-fold higher risk of visual impairment than peripheral retinal damage (54). OT usually presents as posterior uveitis with unilateral choroidal retinopathy and vitritis. Common complications are increased intraocular pressure, cataract, posterior vitreous detachment, retinal detachment, retinal choroidal scarring, and retinal neovascularization (58, 59). Immunocompromised individuals are also more likely to develop OT, with HIV-positive patients having a 2.1 times higher risk of the disease than HIV-negative patients (60).

### 5.2 Clinical manifestations of OT

Retinal choroiditis is the most common feature of active intraocular inflammation in patients with OT. Early fundus fluorescein angiography (FFA) shows inflammation in areas of low fluorescence, followed by progressive leakage from surrounding retinal vessels and margins of the main lesion, resulting in areas of high fluorescence (61). Inflammation in the anterior chamber of the eye can block aqueous outflow channels leading to increased intraocular pressure (IOP), which is associated with increased anterior chamber cellularity and macular involvement (62). Severe vitritis is characterized by a bright white reflex observed by indirect fundoscopy that resembles “headlight in the fog” (59). Vitritis can cause anterior retinal membrane formation and subsequent vitreoretinal traction in the region of retinal choroiditis, leading to complications such as retinal detachment, vitreomacular traction syndrome, and vitreous hemorrhage. Acute OT presents with extensive retinal necrosis and is usually accompanied by vasculitis. It is predominantly characterized by multifocal segmental retinal arteritis (SRA), also known as Kyrieleis arteritis (63). Active lesions are white with blurred borders located near atrophic or hyperpigmented scars (29). During the relapsing phase, the lesions occur near previous scars with varying hyperpigmentation (61). Moreover, *T. gondii* infection also promotes the development of punctate extraretinal toxoplasmosis (PORT), neuroretinitis, retinal vascular occlusion, secondary Coats’ disease, Fuchs’ syndrome, and sclerochoroiditis (64), all of which are less commonly reported or present in particular individuals.

## 6 Diagnostic methods for OT

*T. gondii*-specific IgG antibodies are detected in most typical clinical cases, suggesting previous infection (congenital or acquired). Although a negative result for IgG antibodies almost excludes *T. gondii* infection, false-negative results can be observed in rare cases (65, 66). Laboratory diagnostic methods for systemic toxoplasmosis include polymerase chain reaction (PCR), serologic testing, immunohistochemical identification, *in vitro* culture, and animal inoculation. Rarely, detection of antigens in serum and body fluids, skin tests, and antigen-specific lymphocyte transformation have also been used (67). Detection of specific antibodies in the aqueous and vitreous humor as well as PCR-based assays can be effectively used to diagnose OT (68). In addition, the severity and prognosis of OT can be assessed by imaging tests such as fundus photography, optical coherence tomography (OCT), OCT angiography, ultrasound, confocal scanning laser ophthalmoscopy, FFA and indocyanine green angiography.

## 7 Breakthroughs in treatment modalities

### 7.1 Routine OT treatments

OT is a self-limiting disease that is incurable and prone to recurrence. Retinal choroiditis caused by *T. gondii* usually resolves within 1–2 months (57). The conventional treatment regimen for OT is a combination of pyrimethamine and sulfonamides (sulfadiazine) with or without systemic glucocorticoids (69), which act in different steps of the tetrahydrofolate synthesis pathway to effectively inhibit parasite growth. In particular, pyrimethamine blocks dihydrofolate reductase activity, and sulfadiazine is a competitive inhibitor of p-aminobenzoic acid (57) that negatively impacts nucleic acid synthesis in *T. gondii*. It has been reported that patients treated with glucocorticoids alone have unfavorable outcomes (70).

### 7.2 Alternative OT treatments

Trimethoprime-sulfamethoxazol is a common alternative medication for OT (71, 72) due to its good safety profile, accessibility and affordability. It has been found that trimethoprime-sulfamethoxazol in combination with prednisolone is safer and more effective than conventional OT treatment (73).

Alternative treatments with systemic or local (intravitreal) antibiotics (74, 75) have emerged in recent years. Intravitreal injection of clindamycin and dexamethasone has been shown to achieve effective control of retinal chorioretinitis (76, 77). In addition, antibiotic treatment may reduce the risk of recurrent *T. gondii*-related retinal choroiditis, but there was insufficient evidence to support its benefit in patient outcomes (75).

Two other antiparasitic drugs, atovaquone and azithromycin, have been found to be effective against OT in experimental studies (78) but not in preventing recurrence of retinal chorioretinitis in humans, which may be attributed to the drug resistance in *T. gondii* (79).

### 7.3 Treatment for OT complications

Verteporfin photodynamic therapy (V-PDT) and intravitreal injection of anti-VEGF antibody (bevacizumab) are safe and effective treatments for toxoplasmosis-associated choroidal neovascularization (CNV) in the macula, and the former has shown better results in children (80–82).

### 7.4 Exosome treatment for *T. gondii*-induced uveitis

Bai et al. verified the role of exosomes secreted by mesenchymal stem cells (MSCs) in an experimental rat model of autoimmune uveoretinitis (83). The authors found that periocular injection of hMSCs-derived exosomes reduced leukocyte infiltration in the eye and alleviated uveitis, attenuating harmful Th1 and Th17 cell-driven immune responses (84). Heightened Th1/Th17 immune responses have been reported in the eyes of OT patients (55), and high levels of Th1 cytokines such as IL-2, IFN-γ, IL-6, IL-17, and MCP-1 were detected in the aqueous humor of OT patients (85). IL-17A is highly expressed in the aqueous humor of OT patients (86) and in the retina of *T. gondii*-infected mice (87). The balance between Th17 and Th1 responses (especially IFN-γ) is critical for the outcome of infection. Sauer et al. reported a novel *in vivo* therapeutic approach for inhibiting intraocular inflammation through intravitreal injection of IL-17A monoclonal antibodies (86). Thus, MSCs may serve as a feasible treatment for ocular inflammation caused by T. gondii infection, and hMSCs-derived exosomes have a broad potential for the treatment of retinal chorioretinitis.

### 7.5 TgMyoA as a potential target for toxoplasmosis treatment

The MyoA motility complex of *T. gondii* has long been recognized as an attractive target for drug development (88–90). *T. gondii* motility is driven, at least in part, by this unusual XIVa-like myosin motor protein myosin A (MyoA), which is found only in apicomplexan parasites and a small number of ciliates. Depletion of *T. gondii* MyoA (TgMyoA) leads to significantly reduced parasite motility, host-cell invasion, and host-cell efflux (91). A drug screening study by Kelsen et al. identified KNX-002 as a compound that inhibits TgMyoA ATPase activity and parasite movement, supporting TgMyoA as a druggable target for toxoplasmosis. However, the risk of drug resistance warrants further investigation (92).

## 8 Discussion

Toxoplasmosis is a refractory infectious disease caused by the parasite *T. gondii*. Current treatments are unable to cure toxoplasmosis, and their side effects may exacerbate the health of some patients with poor systemic conditions. Therefore, the development of new, highly effective, and low-toxicity anti-*T. gondii* drugs and preventive vaccines is a key direction of future research. Furthermore, improving the diagnosis and surveillance of toxoplasmosis, interdisciplinary cooperation and public health education are also crucial for controlling the disease and ensuring timely and effective treatment.

*T. gondii* invasion in the eye is complicated by the presence of the BRB and the specialized functions of various retinal cells. This complexity makes it challenging to apply findings from non-ocular models to the pathophysiology of OT. New insights into the immune mechanisms of OT may provide clues for the development of topical medications that can minimize ocular complications and eradicate parasites from the eye to prevent recurrence. Therefore, further studies are warranted to clarify the mechanisms of retinal infiltration and inflammation in OT.

## Author contributions

ZC: Writing – original draft, Writing – review & editing. SC: Writing – review & editing. XC: Writing – review & editing. ZZ: Writing – review & editing. YD: Writing – review & editing.
